# Age-Related Decrease of Meiotic Cohesins in Human Oocytes

**DOI:** 10.1371/journal.pone.0096710

**Published:** 2014-05-07

**Authors:** Makiko Tsutsumi, Reiko Fujiwara, Haruki Nishizawa, Mayuko Ito, Hiroshi Kogo, Hidehito Inagaki, Tamae Ohye, Takema Kato, Takuma Fujii, Hiroki Kurahashi

**Affiliations:** 1 Division of Molecular Genetics, Institute for Comprehensive Medical Science, Fujita Health University, Toyoake, Aichi, Japan; 2 Department of Obstetrics and Gynecology, Fujita Health University, Toyoake, Aichi, Japan; 3 Department of Anatomy and Cell Biology, Gunma University Graduate School of Medicine, Maebashi, Gunma, Japan; University of Science and Technology of China, China

## Abstract

Aneuploidy in fetal chromosomes is one of the causes of pregnancy loss and of congenital birth defects. It is known that the frequency of oocyte aneuploidy increases with the human maternal age. Recent data have highlighted the contribution of cohesin complexes in the correct segregation of meiotic chromosomes. In mammalian oocytes, cohesion is established during the fetal stages and meiosis-specific cohesin subunits are not replenished after birth, raising the possibility that the long meiotic arrest of oocytes facilitates a deterioration of cohesion that leads to age-related increases in aneuploidy. We here examined the cohesin levels in dictyate oocytes from different age groups of humans and mice by immunofluorescence analyses of ovarian sections. The meiosis-specific cohesin subunits, REC8 and SMC1B, were found to be decreased in women aged 40 and over compared with those aged around 20 years (P<0.01). Age-related decreases in meiotic cohesins were also evident in mice. Interestingly, SMC1A, the mitotic counterpart of SMC1B, was substantially detectable in human oocytes, but little expressed in mice. Further, the amount of mitotic cohesins of mice slightly increased with age. These results suggest that, mitotic and meiotic cohesins may operate in a coordinated way to maintain cohesions over a sustained period in humans and that age-related decreases in meiotic cohesin subunits impair sister chromatid cohesion leading to increased segregation errors.

## Introduction

Chromosomal segregation errors that arise during meiotic cell division produce aneuploid gametes with an incorrect number of chromosomes. Aneuploidy of fetal chromosomes causes miscarriage or results in newborns with congenital birth defects such as Down syndrome (DS). It is well known that the extra chromosome in trisomy 21 in DS mainly originates from segregation errors during maternal meiosis I, and that the risk of DS increases with maternal age [1], [2]. The frequency of DS is 1/1400 births in 20–24 year-old women. This rises to 1/350 in 35-year-old and 1/25 in 45-year-old and older women [3]. In addition, age-related oocyte aneuploidy causes an elevated risk of pregnancy loss in older women i.e. the rate of chromosomal anomalies among clinically diagnosed miscarriages is about 50% in women younger than 35 but is 75% in women above this age. The incidence of miscarriage is 9% in women in their early 20 s and rises to 75% in women aged 45 and over [4], [5]. These also indicate significant increase of aneuploidy in older women. However, the etiology of maternal age-related increases in chromosomal segregation errors remains unclear.

Meiotic cohesins have a key role in correct segregation of chromosomes in meiosis [6]–[8]. The ring-shaped cohesin complex is required for sister chromatid cohesion during meiosis and also in mitotic cell division. In mammalian oocytes, the establishment of cohesions during pre-meiotic DNA replication is followed by entry into prophase of meiosis I. After homologous chromosomes are connected at the crossover site via homologous recombination, the oocytes become arrested at the diplotene stage of prophase I for a prolonged period (dictyate stage) during the fetal stage. After sexual maturation, meiosis resumes and the first cell division occurs before ovulation. During this prolonged period, the homologous chromosomes are kept together until the onset of anaphase I by sister chromatid cohesion distal to the chiasma. However, it has been shown that SMC1B and REC8, meiosis-specific components of the cohesin complex, do not undergo turnover after birth in female mice [9], [10]. It is possible that this might cause the gradual decrease in the cohesin levels. The deterioration of cohesion in the chromosome arm leads to meiosis I nondisjunction via the premature dissociation of chiasma between homologous chromosomes. This deterioration in the centromere induces predivision of sister chromatids. These events result increase the risk of age-related segregation error in humans [11]–[14].

To verify this hypothesis, the levels of the meiotic cohesin subunits, SMC1B and REC8, as well as the mitotic cohesin subunits, RAD21 and SMC1A, and the SMC3 subunit used in common between meiotic and mitotic cohesin complexes, was examined in dictyate oocytes from humans and mice. The immunofluorescence signal intensities on ovarian tissue sections were quantified and demonstrated an age-related decrease in the meiosis-specific cohesins in both humans and mice. These results lend support to the hypothesis that a decrease in the cohesins with age induces chromosomal segregation errors. Moreover, these results highlight a novel hypothesis underlying the maintenance mechanism of cohesions in humans in which the life span is much longer than that of mice.

## Materials and Methods

### Ethics statement

This study was approved by the Ethical Review Board for Clinical Research at Fujita Health University. All of the patients gave written informed consent. All animal experiments were approved by the Animal Care and Use Committee at Fujita Health University (I0501).

### Human ovarian tissue

Human ovarian tissues were obtained from 8 women (age range: 19–49 years) who were undergoing surgery for ovarian tumors at Fujita Health University Hospital. Samples were derived from the normal region within the dissected tumor. Ovarian tissues were immediately embedded in Tissue-Tek OCT compound (Sakura Finetek), snap-frozen in liquid nitrogen in the operating room and stored at −70°C within 1 hour of dissection.

### Mice

C57BL/6NCr mice were obtained from Japan SLC. Ovaries from female mice were embedded, snap-frozen and stored at −70°C as described above.

### Antibodies

Rabbit polyclonal anti-human REC8 antibodies were raised against amino acid residues 427–443 of the human REC8 protein. Rabbit polyclonal anti-human SMC1B antibodies were raised against amino acid residues 1219–1235 of the human SMC1B protein. Guinea pig polyclonal anti-mouse SMC1B antibodies were raised against amino acid residues 1215–1248 of mouse SMC1B. Guinea pig polyclonal anti-mouse REC8 antibodies were raised as described previously [15]. The resulting antisera were affinity purified on columns coupled to the peptide. The specificities of the antibodies described above were confirmed as shown in Figure S1. All antibodies used in this study are listed in Table S1.

### Immunofluorescent staining

Ovarian frozen tissue sections were prepared and fixed as described previously [16]. Briefly, sections were fixed in methanol:acetone:chloroform (1∶1∶1) for 10 min on ice and then washed three times in PBS. After blocking with 10% normal donkey serum in PBS for 30 min at room temperature, staining with primary and secondary antibodies were performed as described [17]. The exception was when using biotin-conjugated secondary antibodies where the slides were incubated with Alexa Fluor 350-conjugaed streptavidin (Life Technologies) for 15 min and then washed three times after washing of the secondary antibodies.

### Image analysis

Slides were observed under a fluorescence microscope (Axio Imager M1 or Axio Imager Z2, Carl Zeiss) equipped with a digital camera (AxioCam HRc, Carl Zeiss). Images were captured using the same settings controlled by Axiovision 4.8 software (Carl Zeiss). Densitometric analysis of the immunofluorescence signals from oocyte and somatic cell nuclei was performed using Axiovision 4.8 software. Schematic drawings explaining the method to determine the relative cohesin concentrations are described in Figure S2. Data analysis was performed using Excel software.

## Results

### Age-related decrease of meiosis-specific cohesins in human dictyate oocytes

**Figure 1:**
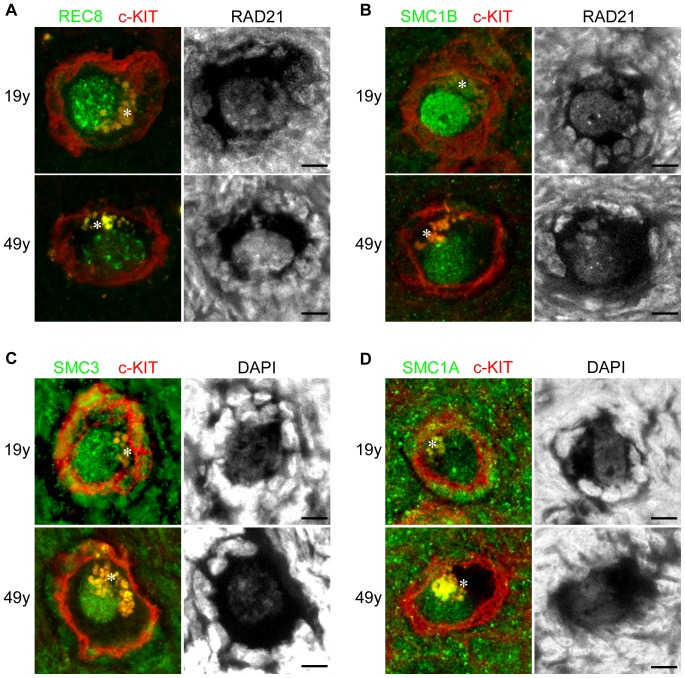
Immunofluorescent staining of human oocytes in ovarian sections from 19- and 49-year-old women. (A, B) REC8 or SMC1B (green) proteins co-immunostained with RAD21. (C, D) SMC3 or SMC1A (green) signals counterstained with DAPI. C-KIT (red) was used as a marker of oocytes. Asterisks indicate autofluorescence in the cytoplasmic region of the oocytes. Bar, 10 µm.

To compare the cohesin levels in oocytes from women of different ages, we employed an immunofluorescence technique using ovarian tissue sections (Fig. 1). Cohesin complexes in oocytes consist of REC8, SMC1B and SMC3 subunits, whilst in somatic cells comprise RAD21, SMC1A and SMC3 subunits. By immunofluorescence staining, meiosis-specific cohesin subunits, REC8 and SMC1B, were detectable in the nuclei of dictyate oocytes and exhibited a threadlike distribution with dense aggregates as previously reported [18]. but not in somatic cells on ovarian tissue sections (Fig. 1A, B). SMC3, a shared cohesin subunit both meiotic and mitotic cells, could be detected in the nuclei of both oocytes and somatic cells (Fig. 1C). The mitotic cohesin subunits RAD21 and SMC1A, which are substituted in meiosis with meiosis-specific cohesins REC8 and SMC1B, respectively, were also detectable both in somatic cells and oocytes (Fig. 1A, B, D).

To determine the relative oocyte cohesin concentration, the immunofluorescent signals corresponding to mitotic cohesins in the nuclei of somatic cells were used to normalize the signal intensities among samples. REC8 or SMC1B proteins were immunostained along with RAD21 as an internal control (Fig. 1A, B). The relative amounts of cohesins in oocytes were estimated as a ratio of the signal intensity in oocytes to that of mitotic cohesins in somatic cells (Fig. S2).

**Figure 2:**
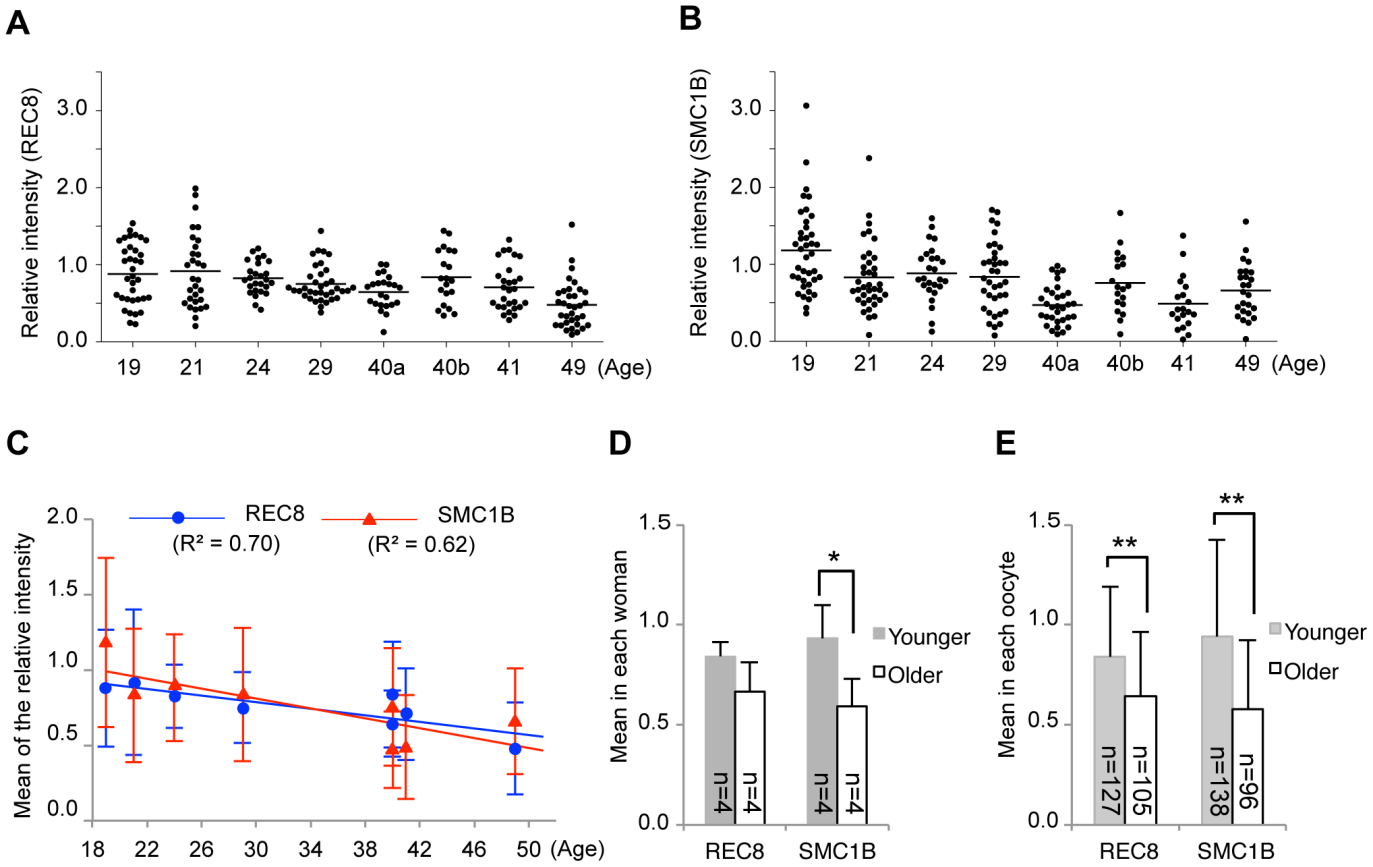
Quantitative results for meiosis-specific cohesins in human oocytes. (A, B) Relative signal intensity of REC8 or SMC1B. Intensities were determined as described in Figure S2. Specimens were obtained from two 40 year old female subjects (40a and 40b). Horizontal bars indicate the mean cohesin levels for each subject. (C) Regression analyses of the mean cohesin signal intensities shown in (A) and (B) (mean ± SD). The regression lines are indicated by solid lines. The coefficients of determination are indicated in parentheses. (D, E) Comparisons of the signal intensity means between grouped samples. Women were grouped as younger (≤29-year-old) or older (≥40-year-old). (D) The cohesin signal intensity means shown in (A–C) were compared between groups (mean ± SD). (E) The cohesin signal intensity means in single oocytes were compared between groups (mean ± SD). *P<0.05, **P<0.01, Student's *t*-test.

We examined the signal intensity of meiosis-specific cohesins in oocytes from 8 women aged 19 to 49 years (Fig. 2A, B). Immunofluorescent signals for the meiosis-specific cohesins, REC8 and SMC1B, were detected in the oocytes of all samples examined (Fig. 1A, B). These signal intensities showed a wide distribution, which was partly due to the variability between each oocyte and also due to the position of the sections since the thicknesses of these sections are smaller than the diameter of the oocyte nuclei (Fig. 1A, B). We found that the mean REC8 and SMC1B signal intensities among individual women showed a negative correlation with age (Fig. 2C). We further analyzed these signal intensities by dividing our female subject into younger (≤29-year-old; mean ± SD: 23.3±4.3 year-old) and older (≥40-year-old; mean ± SD: 42.5±4.4 year-old) groups. The decrease observed in the SMC1B levels in the older group was significant although REC8 did not show a significant decrease (Fig. 2D). However, when the signal intensities of each oocyte were compared, the REC8 and SMC1B levels in the older group were significantly decreased by 24% and 38%, respectively, compared with the younger women (Fig. 2E). Similar results were obtained when REC8 was co-immunostained with pan-histone as another internal control (Fig. S3).

**Figure 3:**
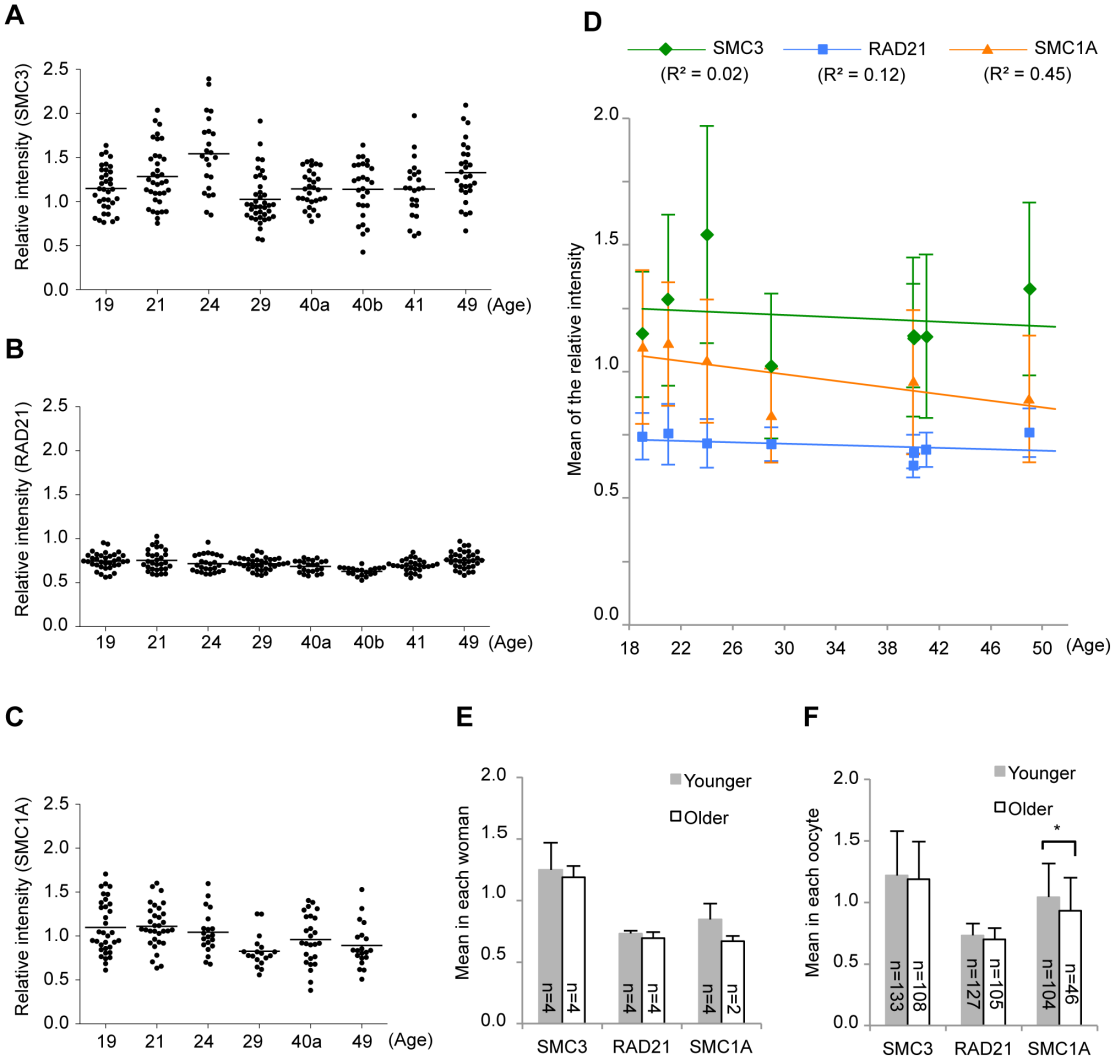
Quantitative results for the mitotic cohesin levels in human oocytes. (A–C) Relative signal intensities for SMC3, RAD21 or SMC1A. (D) Regression analyses of the cohesin signal intensity means shown in (A–C) (mean ± SD). Lines are the regression lines. The coefficients of determination are in parentheses. (E, F) Comparisons of the signal intensity means between grouped samples. Women were grouped as indicated in Figure 2. (E) The cohesin signal intensity means shown in (A–D) were compared between groups (mean ± SD). (F) The cohesin signal intensity means in single oocytes were compared between groups (mean ± SD). *P<0.05, Student's *t*-test.

We also examined the relative SMC3, RAD21 and SMC1A levels in human oocytes (Figs. 1 and 3A–C). In contrast to the meiosis-specific cohesins, the signal intensities of these proteins did not significantly change with age, although a slight decrease in the SMC1A level was evident in oocytes of older women (Fig. 3D–F).

### Cohesins in the dictyate oocytes of the mouse

**Figure 4:**
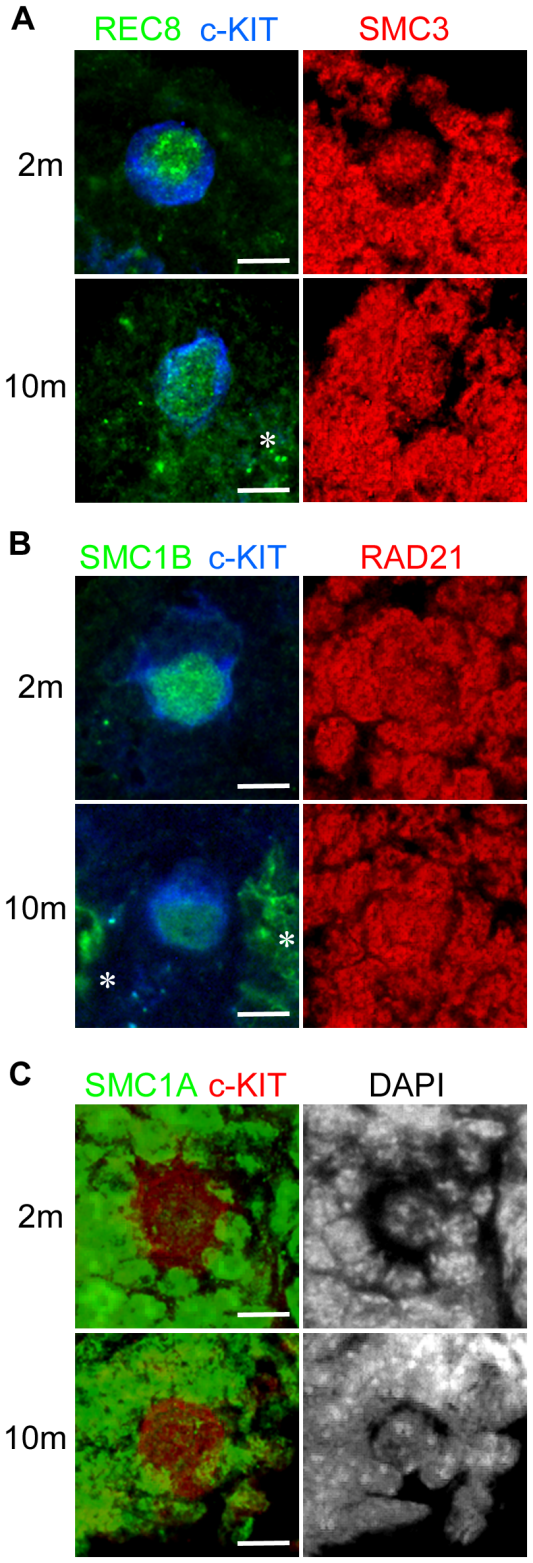
Immunofluorescent staining of mouse oocytes in ovarian sections from 2- and 10-month-old female mice. (A) Slides were co-immunostained with REC8 (green) and SMC3 (red). (B) Slides were co-immunostained with SMC1B (green) and RAD21 (red). (C) SMC1A (green) signals with DAPI nuclear counterstaining. Asterisks indicate autofluorescence. Bar, 10 µm.

**Figure 5:**
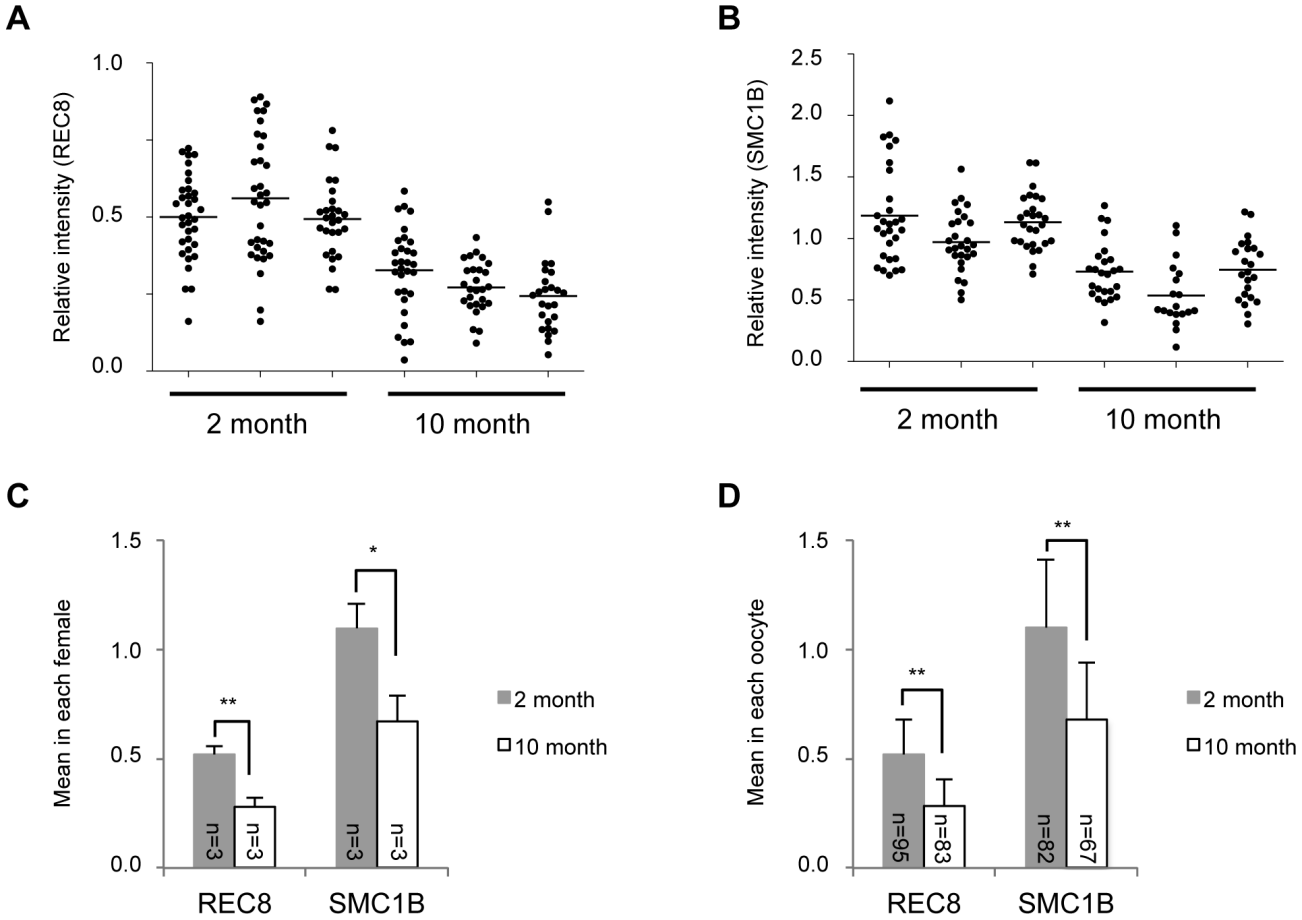
Quantitative results for meiosis-specific cohesin levels in 2- and 10-month-old mouse oocytes. (A, B) Relative signal intensity of REC8 or SMC1B. Intensities were determined as indicated in Figure S2. Three mice were used at each age. Horizontal bars are the mean cohesin levels within each female. (C) Cohesin signal intensity means in each female (mean ± SD). (D) Cohesin signal intensity means in each oocyte (mean ± SD). *P<0.05, **P<0.01, Student's t-test.

To evaluate whether our above findings are unique to humans in which a long life span leads to years of dictyate arrest, we performed similar immunofluorescence experiments in the mouse. Two-month-old and 10-month-old wild-type B6 mice, which have just become sexually matured and have reached the late reproductive age, respectively, were examined (Fig. 4). In each age group, samples were derived from 3 mice. The signal intensity of REC8 in the older mice was significantly lower than that of younger mice (Fig. 5A, C, D) as demonstrated in our human analyses (Fig. 2). Likewise, the signal intensity of SMC1B in older mice was also low relative to that of younger mice (Fig. 5B, C, D). Thus, meiosis-specific cohesins in mouse oocytes decrease in an age-related manner as with humans.

**Figure 6:**
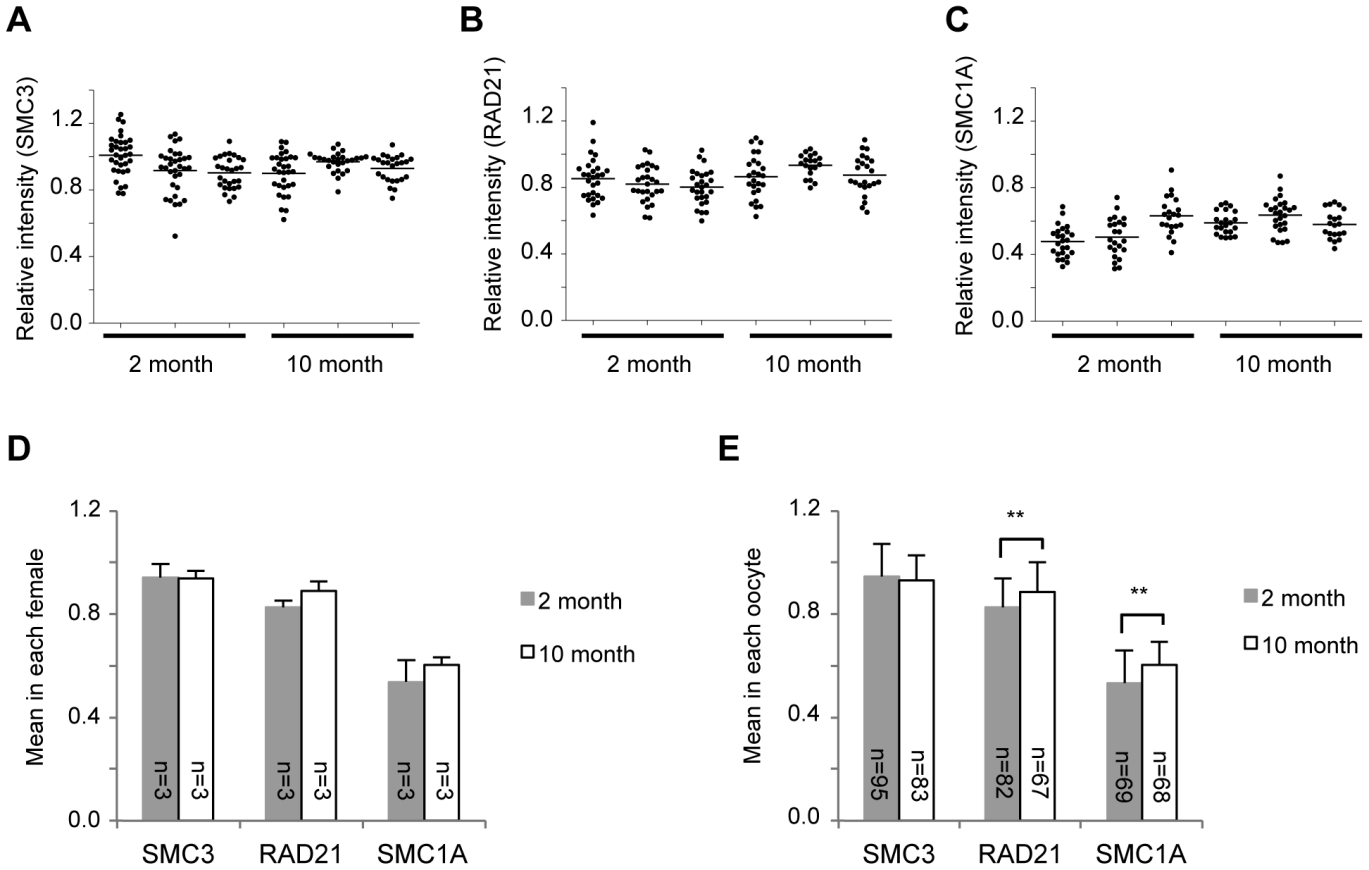
Quantitative results for mitotic cohesin levels in mouse oocytes. (A–C) Signal intensities for SMC3, RAD21 or SMC1A determined as described in Figure S2. (D) Cohesin signal intensity means in each female (mean ± SD). (E) Cohesin signal intensity means in each oocyte (mean ± SD). **P<0.01, Student's *t*-test.

We found from our immunofluorescence analyses that the signal intensity of SMC3 in older mice was not significantly different from that of younger mice. This result is similar to that found in humans, although the signal intensities of oocytes to somatic cells in mice were found to be slightly lower than those in humans (Fig. 6A, D, E). In contrast, the signal intensity of RAD21 in older mice showed a slight increase compared with younger mice (Fig. 6B, D, E). Interestingly, and in contrast to humans, the immunofluorescent signal intensity of SMC1A in the nuclei of mouse oocytes was barely detectable relative to that in somatic cells (Figs. 1D and 4C). Although the mean signal intensity of SMC1A in each older mouse was almost similar to that in each younger mouse (Fig. 6C, D), the mean signal intensity in each oocyte was increased slightly in older mice, as was observed for RAD21 (Fig. 6E).

## Discussion

### Age-related decrease in meiosis-specific cohesin subunits

In our present study, we demonstrate an age-related decrease of meiosis- specific cohesin subunits, REC8 and SMC1B, in dictyate oocytes both in humans and mice. In contrast, the signal levels of the cohesin subunits common to mitotic cells, SMC3, RAD21 and SMC1A, did not change. To our knowledge, this is the first report to demonstrate an age-related decrease in the cohesin concentrations in human oocytes at the protein level. Although it has been reported that cohesin staining in MI and MII oocytes from women of different ages (age range: 18–34) indicated no decrease in the older women [18], this may be possibly attributed to the samples derived from relatively young women compared to our present study.

It has been long accepted that age-related increases in oocyte aneuploidy do not occur in mice. Long dictyate arrest in humans due to a long life span is likely to underlie this difference. However, age-related aneuploidy has been detected in certain mouse strains such as C57BL/6 [19], [20]. Indeed, age-related decreases in REC8 have been reported previously in the C57BL-related strains [11], [12]. It is therefore currently believed that this phenomenon is strain-dependent. These findings are consistent with the linkage between the age–related increase of aneuploidy and decrease in cohesin levels seen also in humans.

The mechanism of reduction in cohesin levels on the chromatin in dictyate oocytes remains to be clarified. Oxidative damage or spontaneous hydrolysis of peptide bonds that are generally involved in protein aging are one possible cause of this degradation [13]. It has also been suggested that leaky separase activity may cause a loss of cohesins [13], [21]. Although separase intrinsically cleaves the cohesin ring at anaphase onset [22]. the expression level of this enzyme was previously found to be up-regulated in metaphase II oocytes in older women compared with younger [23].

The expression patterns of cohesin mRNAs in human oocytes determined by microarray analyses were reported previously [23], [24]. Whilst SMC3, RAD21 and SMC1A mRNAs are substantially expressed in oocytes, REC8 and SMC1B transcripts were only weakly detectable in these experiments. Thus, once meiosis-specific cohesin subunits undergo degradation, they would not undergo turnover in human or mouse, which facilitates the age-related decrease in the meiotic cohesin levels [9], [10].

### Coordination of meiotic and mitotic cohesins

Whilst the meiotic cohesins in mouse oocytes of a certain strain decrease within a few months, the cohesins are retained for decades in humans. It is tempting to imagine that special safeguarding mechanism had to be evolved in the long-living humans, and those mechanisms are absent in mice. We speculate that mitotic and meiotic cohesins may coordinately contribute maintaining the cohesion levels for long periods in humans. In our current study, we show that the SMC1A level was comparable between oocytes and somatic cells in humans, but only marginally detectable in mice. Based on the amino acid sequence of its epitope, the anti-SMC1A antibody used for detection of human SMC1A will not cross react with human SMC1B which is present in oocytes. It is suggested that both SMC1A-RAD21 and SMC1B-REC8 cohesins are present in human oocytes, and that SMC1A-RAD21 undergoes turnover even during dictyate arrest. This might partly contribute to maintaining the cohesin levels for a prolonged period in humans. In addition, the signal intensities of RAD21 and SMC1A were found to be slightly increased in aged mice in our present experiments. We suggest that this is supportive evidence for the upregulation of the mitotic cohesins in mouse oocytes to compensate for the loss of meiosis-specific cohesins. However, it has been shown that the SMC1A level is too marginal to compensate for the reduction in SMC1B in the mouse [25]. Nonetheless, no increase in SMC1A or RAD21 has been observed in human in accordance with the decrease in their meiotic counterparts, SMC1B and REC8. Even the baseline levels of SMC1A and RAD21 in human are still not sufficient to completely compensate for the loss of meiosis-specific cohesins. It is possible in this regard that the newly synthesized cohesins may be less able to hold sister chromatids together during meiosis since the cohesion establishment factors are not recruited without the DNA replication machinery [26], [27].

### Age-related decrease in cohesin and increase in aneuploidy

The frequency of trisomy in conceptuses is 2–3% in women aged in their twenties, but it rises exponentially in women in their mid-thirties to reach 35% in women aged over 40 [1]. However, the meiosis-specific cohesin levels in our current experiments showed a linear negative correlation with the age of the sample donors. As a cause of this disparity, one possibility is a threshold effect of the cohesin level with regard to segregation error susceptibility i.e. there may be a threshold level of cohesins that can hold sister chromatids together against physical force. We thus speculate that the meiotic chromosomes may undergo malsegregation until these levels reach this threshold.

An alternative possibility is that the cohesins, in regulating gene expression, are involved in the exponential increase in aneuploidy. Cohesin depletion may cause a substantial effect on gene expression through defects in transcriptional regulatory functions [8], [28], [29]. Indeed, the expression of various genes in mature oocytes has been shown to change with age [23], [30], [31]. Thus, the chromosomes with impaired cohesions would be more susceptible to segregation error possibly because of spindle checkpoint defects caused by altered gene expression [23], [32].

In accordance with the results of our present study, the age-related increase of interkinetochore distance between sister chromatids has been observed in the metaphase II oocytes of humans, suggesting a deterioration of chromosome cohesion with advancing age [33]. Without replenishment of meiosis-specific cohesins, cohesion between sister chromatids gradually deteriorates over time. This causes premature resolution of the chiasma between homologs or the premature separation of sister chromatids during the dictyate stage. Since these pre-separated chromosomes are distributed into daughter cells randomly, they are prone to malsegregation leading to gametes with aneuploidy. In our present study, besides the age-effect, we observed a variation among individuals in the rate of cohesin decrease. This might be due to the differences in genetic background and/or lifestyle. In future studies, a determination of the genetic variation affecting cohesin robustness will help to predict the susceptibility of individuals. A further elucidation of the mechanism underlying the decrease or maintenance of meiotic cohesins will contribute to the development of novel strategies to prevent age-related aneuploidy.

## Supporting Information

Figure S1 **Specificity of the antibodies.** (A) Western blot analysis using affinity-purified antibodies. Testes lysates from human or mouse were loaded on the same gel. After electrophoresis and blotting, blots were stained with Ponceau S, and the each lane was cut into strips. SMC1B and REC8 were detected as 145-kDa and 75- to 82-kDa bands, respectively. Asterisks and dots indicate nonspecific bands reacted with the secondary antibodies. (B) Negative control of cohesin-immunostaining using IgGs and the secondary antibodies. (C) Negative control of cohesin-immunostaining using preimmune sera. (D) Negative control of cohesin-immunostaining using antibodies absorbed by preincubation with the peptide immunogens.
(PDF)

Figure S2 **Representative immunofluorescent staining pattern of human oocytes.** Green and blue signal intensities within the circled areas, indicating the oocyte nucleus, were respectively determined, as well as 5 randomly chosen somatic nuclei in the vicinity of the oocyte. The signal intensity of REC8 (green) was defined as: (area density of green signal in oocyte nucleus - mean area density of green signal in 5 somatic nuclei)/mean area density of blue staining in 5 somatic nuclei. Because the green signals detected in somatic nuclei are background, these were subtracted from the signals in the oocytes. The signals in the oocytes were adjusted using the blue signal corresponding to the RAD21 cohesin subunit that is constitutively expressed in somatic cells. The signal intensity for RAD21 was defined as: area density of blue signal in the oocyte nucleus/mean area density of blue signal in 5 somatic nuclei.
(PDF)

Figure S3 **Quantitative results for REC8 in human oocytes adjusted for the histone signals of somatic nuclei.** (A) Immunofluorescent staining of human oocytes in ovarian sections from 19- and 49- year-old women. REC8 (green) proteins co-immunostained with histone (red) using anti-histone antibody (1∶400; H11-4; Millipore). C-KIT (blue) was used as a marker of oocytes. Asterisks indicate autofluorescence in the cytoplasmic region of the oocytes. Bar, 10 µm. (B) Relative signal intensity of REC8. Intensities were determined as described in Figure S2, more specifically, histone signal in somatic nuclei was used to adjust the REC8 signal intensity. Specimens were obtained from 4 women (age range: 19–49 years). (C) Regression analysis of the REC8 signal intensity means shown in (B) (mean ± SD). Line indicates the regression line. The coefficient of determination is in parenthesis. (D) Comparisons of the signal intensity means between grouped samples. Women were grouped as indicated in Figure 2. The REC8 signal intensity means in single oocytes were compared between groups (mean ± SD). **P<0.01, Student's *t*-test.
(PDF)

Table S1 **Antibodies used in this study.**
(PDF)
