# Supplementary material for: Age-Related Decrease of Meiotic Cohesins in Human Oocytes
Source: PLoS One. 2014 May 7;9(5):e96710. doi: 10.1371/journal.pone.0096710 (PMC4013030; doi:10.1371/journal.pone.0096710)
Supplement: Figure S1 — Specificity of the antibodies. (A) Western blot analysis using affinity-purified antibodies. Testes lysates from human or mouse were loaded on the same gel. After electrophoresis and blotting, blots were stained with Ponceau S, and the each lane was cut into strips. SMC1B and REC8 were detected as 145-kDa and 75- to 82-kDa bands, respectively. Asterisks and dots indicate nonspecific bands reacted with the secondary antibodies. (B) Negative control of cohesin-immunostaining using IgGs and the secondary antibodies. (C) Negative control of cohesin-immunostaining using preimmune sera. (D) Negative control of cohesin-immunostaining using antibodies absorbed by preincubation with the peptide immunogens. (PDF) [file pone.0096710.s001.pdf]

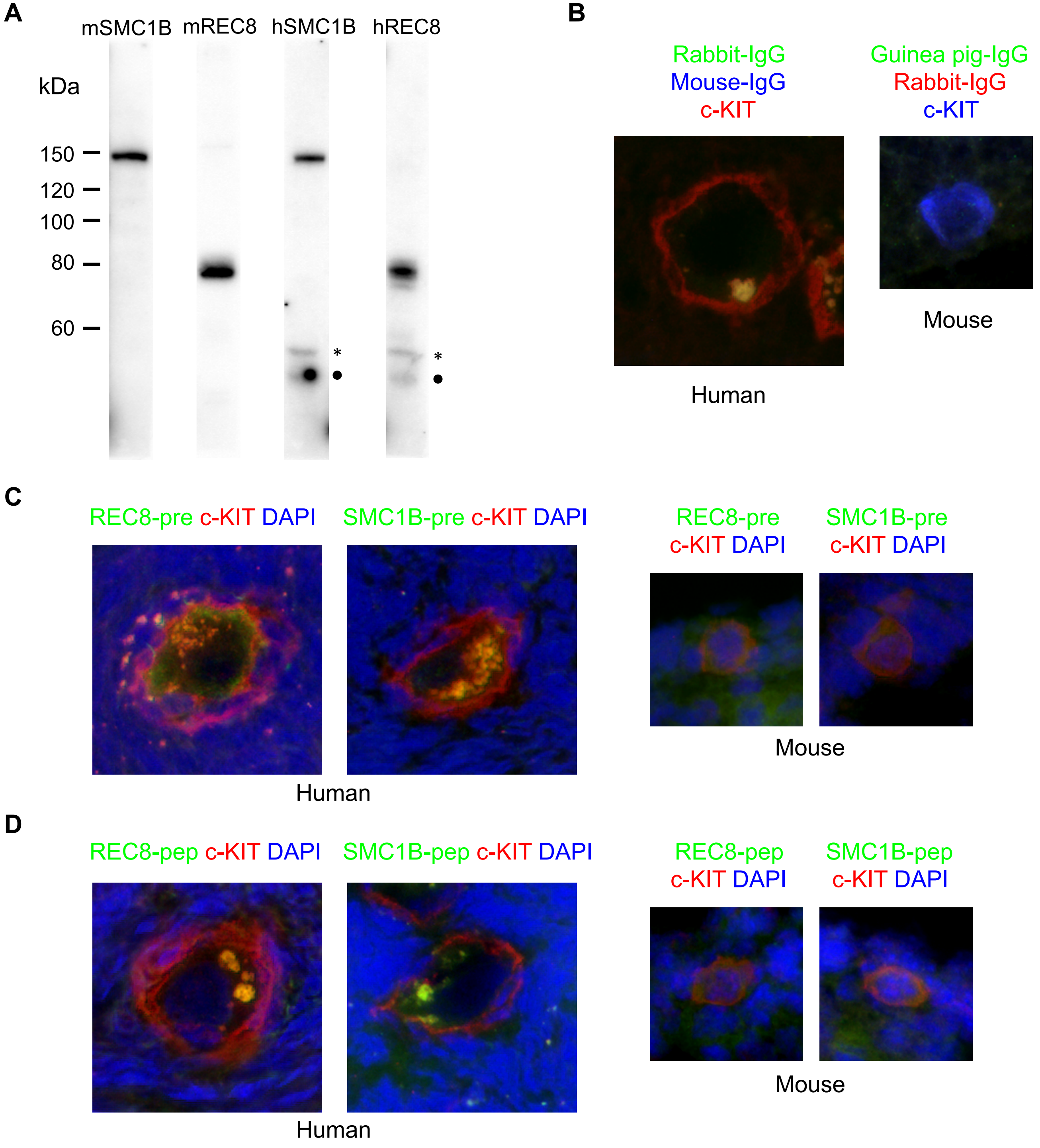

Figure S1. Specificity of the antibodies. (A) Western blot analysis using affinity-purified antibodies. Testes lysates from human or mouse were loaded on the same gel. After electrophoresis and blotting, blots were stained with Ponceau S, and the each lane was cut into strips. SMC1B and REC8 were detected as 145-kDa and 75- to 82-kDa bands, respectively. Asterisks and dots indicate nonspecific bands reacted with the secondary antibodies. (B) Negative control of cohesin-immunostaining using IgGs and the secondary antibodies. (C) Negative control of cohesin-immunostaining using preimmune sera. (D) Negative control of cohesin-immunostaining using antibodies absorbed by preincubation with the peptide immunogens.
