# Supplementary material for: Age-Related Decrease of Meiotic Cohesins in Human Oocytes
Source: PLoS One. 2014 May 7;9(5):e96710. doi: 10.1371/journal.pone.0096710 (PMC4013030; doi:10.1371/journal.pone.0096710)
Supplement: Figure S2 — Representative immunofluorescent staining pattern of human oocytes. Green and blue signal intensities within the circled areas, indicating the oocyte nucleus, were respectively determined, as well as 5 randomly chosen somatic nuclei in the vicinity of the oocyte. The signal intensity of REC8 (green) was defined as: (area density of green signal in oocyte nucleus - mean area density of green signal in 5 somatic nuclei)/mean area density of blue staining in 5 somatic nuclei. Because the green signals detected in somatic nuclei are background, these were subtracted from the signals in the oocytes. The signals in the oocytes were adjusted using the blue signal corresponding to the RAD21 cohesin subunit that is constitutively expressed in somatic cells. The signal intensity for RAD21 was defined as: area density of blue signal in the oocyte nucleus/mean area density of blue signal in 5 somatic nuclei. (PDF) [file pone.0096710.s002.pdf]

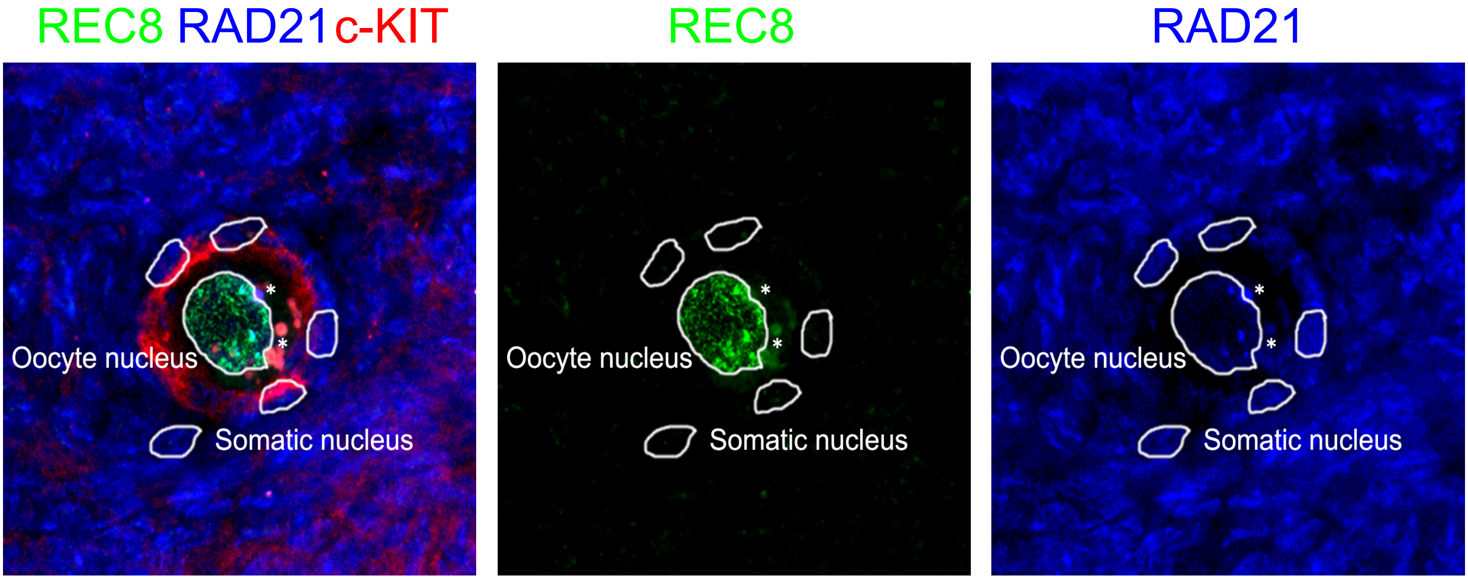

Figure S2. Representative immunofluorescent staining pattern of human oocytes. Green and blue signal intensities within the circled areas, indicating the oocyte nucleus, were respectively determined, as well as 5 randomly chosen somatic nuclei in the vicinity of the oocyte. The signal intensity of REC8 (green) was defined as: (area density of green signal in oocyte nucleus - mean area density of green signal in 5 somatic nuclei) / mean area density of blue staining in 5 somatic nuclei. Because the green signals detected in somatic nuclei are background, these were subtracted from the signals in the oocytes. The signals in the oocytes were adjusted using the blue signal corresponding to the RAD21 cohesin subunit that is constitutively expressed in somatic cells. The signal intensity for RAD21 was defined as: area density of blue signal in the oocyte nucleus / mean area density of blue signal in 5 somatic nuclei.
