# Supplementary material for: Age-Related Decrease of Meiotic Cohesins in Human Oocytes
Source: PLoS One. 2014 May 7;9(5):e96710. doi: 10.1371/journal.pone.0096710 (PMC4013030; doi:10.1371/journal.pone.0096710)
Supplement: Figure S3 — Quantitative results for REC8 in human oocytes adjusted for the histone signals of somatic nuclei. (A) Immunofluorescent staining of human oocytes in ovarian sections from 19- and 49- year-old women. REC8 (green) proteins co-immunostained with histone (red) using anti-histone antibody (1∶400; H11-4; Millipore). C-KIT (blue) was used as a marker of oocytes. Asterisks indicate autofluorescence in the cytoplasmic region of the oocytes. Bar, 10 µm. (B) Relative signal intensity of REC8. Intensities were determined as described in Figure S2, more specifically, histone signal in somatic nuclei was used to adjust the REC8 signal intensity. Specimens were obtained from 4 women (age range: 19–49 years). (C) Regression analysis of the REC8 signal intensity means shown in (B) (mean ± SD). Line indicates the regression line. The coefficient of determination is in parenthesis. (D) Comparisons of the signal intensity means between grouped samples. Women were grouped as indicated in Figure 2. The REC8 signal intensity means in single oocytes were compared between groups (mean ± SD). **P<0.01, Student's t-test. (PDF) [file pone.0096710.s003.pdf]

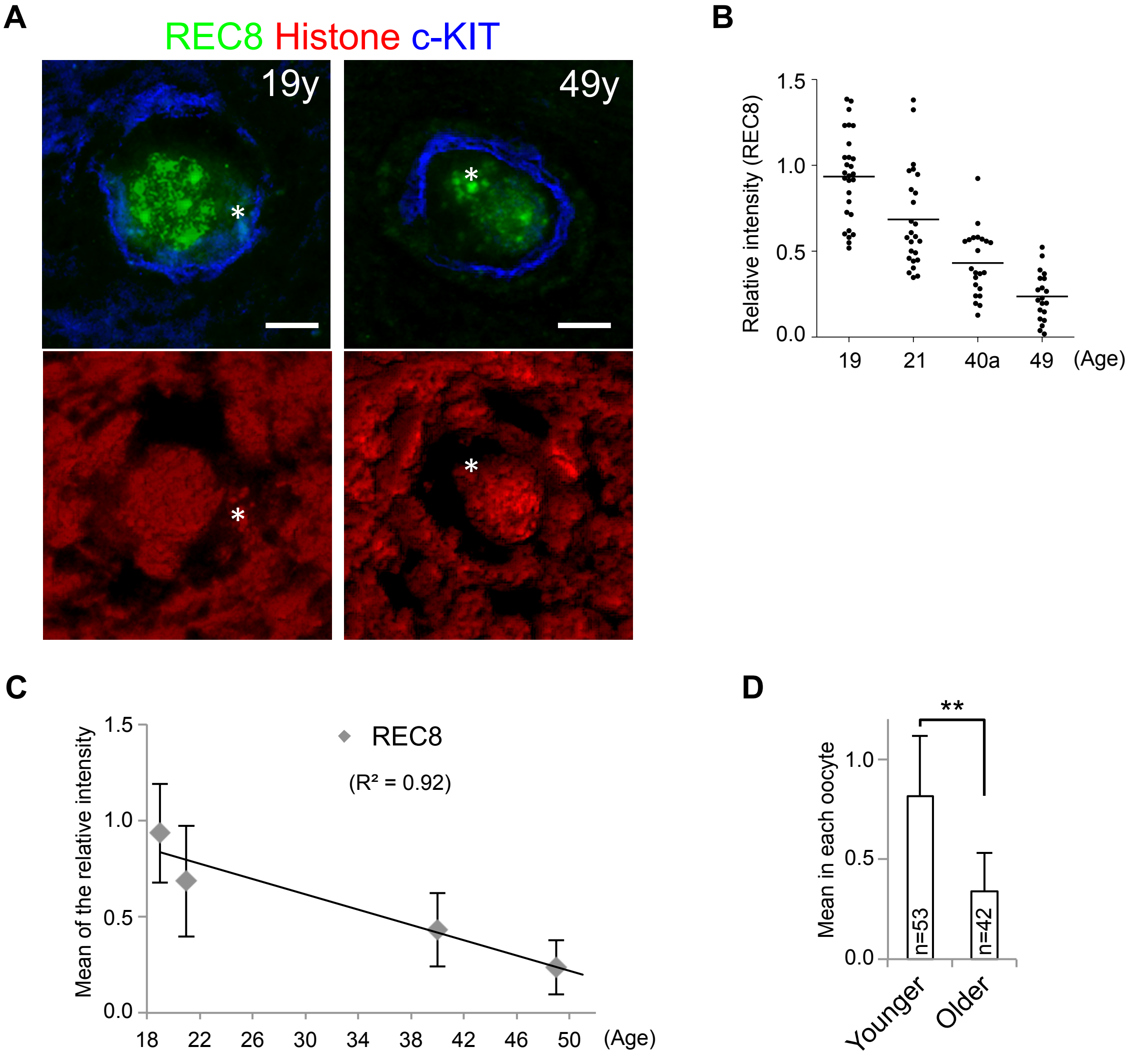

Figure S3. Quantitative results for REC8 in human oocytes adjusted for the histone signals of somatic nuclei. (A) Immunofluorescent staining of human oocytes in ovarian sections from 19- and 49-year-old women. REC8 (green) proteins co-immunostained with histone (red) using anti-histone antibody (1:400; H11-4; Millipore). C-KIT (blue) was used as a marker of oocytes. Asterisks indicate autofluorescence in the cytoplasmic region of the oocytes. Bar, 10 µm. (B) Relative signal intensity of REC8. Intensities were determined as described in Figure S2, more specifically, histone signal in somatic nuclei was used to adjust the REC8 signal intensity. Specimens were obtained from 4 women (age range: 19-49 years). (C) Regression analysis of the REC8 signal intensity means shown in (B) (mean ± SD). Line indicates the regression line. The coefficient of determination is in parenthesis. (D) Comparisons of the signal intensity means between grouped samples. Women were grouped as indicated in Figure 2. The REC8 signal intensity means in single oocytes were compared between groups (mean ± SD). $^{**}P < 0.01$, Student's *t*-test.
