# Supplementary material for: Age-Related Decrease of Meiotic Cohesins in Human Oocytes
Source: PLoS One. 2014 May 7;9(5):e96710. doi: 10.1371/journal.pone.0096710 (PMC4013030; doi:10.1371/journal.pone.0096710)
Supplement: Table S1 — Antibodies used in this study. (PDF) [file pone.0096710.s004.pdf]

Table S1 Antibodies used in this study

Antibody	Source	Company	Dilution
hREC8	Rabbit		1:1000
mREC8	Guinea pig		1:100
hSMC1B	Rabbit		1:200
mSMC1B	Guinea pig		1:5000
SMC1A[a] (AB-957)	Rabbit	SIGMA	1:100
SMC1[b] (ab21583)	Rabbit	Abcam	1:1000
Rad21[c] (53A303)	Mouse	Millipore	1:400
Rad21[d] (ab42522)	Rabbit	Abcam	1:1000
SMC3 (A300-060A)	Rabbit	Bethyl Laboratories	1:500
c-Kit (M-14)	Goat	Santa Cruz Biotechnology	1:400
Alexa Fluor 488-conjugated anti-rabbit IgG	Donkey	Life Technologies	1:200-1000
Alexa Fluor 594-conjugated anti-rabbit IgG	Donkey	Life Technologies	1:200
Alexa Fluor 594-conjugated anti-goat IgG	Donkey	Life Technologies	1:1000
FITC-conjugated anti-guinea pig IgG	Donkey	Jackson ImmunoResearch	1:100
AMCA-conjugated anti-mouse IgG	Donkey	Jackson ImmunoResearch	1:100
Biotin-SP-conjugated anti-goat IgG	Donkey	Jackson ImmunoResearch	1:200

[a]For detection of hSMC1A.

[b]For detection of mSMC1A.

[c]For detection of hRAD21.

[d]For detection of mRAD21.
